# A review of common statistical methods for dealing with multiple pollutant mixtures and multiple exposures

**DOI:** 10.3389/fpubh.2024.1377685

**Published:** 2024-05-09

**Authors:** Guiming Zhu, Yanchao Wen, Kexin Cao, Simin He, Tong Wang

**Affiliations:** ^1^Department of Health Statistics, School of Public Health, Shanxi Medical University, Taiyuan, China; ^2^Key Laboratory of Coal Environmental Pathogenicity and Prevention (Shanxi Medical University), Ministry of Education, Taiyuan, China

**Keywords:** health effects, epidemiology, statistical methods, multi-pollutant mixtures, environment

## Abstract

Traditional environmental epidemiology has consistently focused on studying the impact of single exposures on specific health outcomes, considering concurrent exposures as variables to be controlled. However, with the continuous changes in environment, humans are increasingly facing more complex exposures to multi-pollutant mixtures. In this context, accurately assessing the impact of multi-pollutant mixtures on health has become a central concern in current environmental research. Simultaneously, the continuous development and optimization of statistical methods offer robust support for handling large datasets, strengthening the capability to conduct in-depth research on the effects of multiple exposures on health. In order to examine complicated exposure mixtures, we introduce commonly used statistical methods and their developments, such as weighted quantile sum, bayesian kernel machine regression, toxic equivalency analysis, and others. Delineating their applications, advantages, weaknesses, and interpretability of results. It also provides guidance for researchers involved in studying multi-pollutant mixtures, aiding them in selecting appropriate statistical methods and utilizing R software for more accurate and comprehensive assessments of the impact of multi-pollutant mixtures on human health.

## Graphical abstract

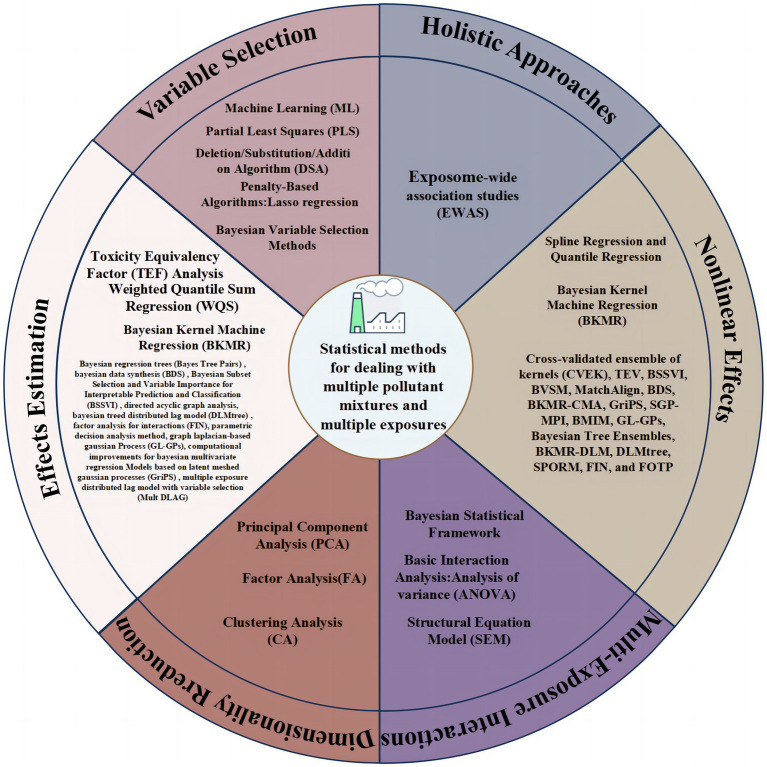

## 1 Introduction

In the contemporary industrialized society, environmental concerns such as air pollution, water pollution, and soil contamination have gained significant attention (1–4). Some pollutants are metabolized because of their shorter half-lives, others, such as heavy metals, insecticides, flame retardants, persistent organic pollutants, and other endocrine-disrupting chemicals continue to accumulate in the human body and have significant and long-term effects on human health (5–8). For instance, the association between heavy metals toxicity and the development of neurodegenerative diseases and various ocular pathologies has been established, while concurrent exposure to heavy metals can elevate the risk of prostate cancer and thyroid enlargement (9–11). Polybrominated diphenyl ether is a persistent and pervasive environmental pollutant that disrupts the human endocrine system, leading to health implications such as developmental, thyroidal, and reproductive toxicity (12, 13). Particulate matter along with nitrogen oxides in the atmospheric environment exhibit a close correlation with stroke incidence rate and mortality. The higher the concentration of particulate matter exposure, the greater risk of stroke (14). However, most studies are on single pollutants, they do not accurately reflect the real world since people are exposed to a combination of several dangerous substances at any given time, which might have antagonistic or synergistic effects. What’s more, single-pollutant analysis methods often fall short in capturing the complexity and interactive effects of multi-pollutant mixtures (15). Lastly, the potential for spurious associations has increased in single-pollutant models, contributing to disagreements between studies. Consequently, accurately assessing the health effects of exposure to mixtures of environmental pollutants has become a focal point in current environmental epidemiology. In recent years, the focus of health effect assessments of environmental risk factors has shifted from the traditional single-pollutant approach to the study of mixtures of multiple pollutants (16–18). This shift aims to more accurately reflect the impact of environmental risk factors on human health.

In order to handle the multi-pollutant mixtures exposure, researchers have raised a number of problems that need to be addressed, as shown in Table 1.

**Table 1:** Key research questions on multi-pollutant mixtures exposure.

Authors or projects	Research questions
Kortenkamp (18, 19)	Overall effects of mixtures, rather than single effect.
Ghassan (16) and Braun (17)	Overall effects of mixtures; Weighting and effects of mixtures; Independent effects of each component of the mixture; Joint effects of each component of the mixture.
Gibson (20)	Are specific exposure patterns present in the study population? What toxic substances are present in the mixture? Alternatively, what is the independent impact of each mixture member on the health outcomes of interest? Are there synergistic effects or interactions among mixture members? What is the overall impact of the mixture on the outcomes of interest?
Powering Research through Innovative methods for Mixtures in Epidemiology (PRIME) program (15)	Overall effect estimation: What is the overall effect of the mixture, and what is the magnitude of the association? Toxin identification: Which congeners/exposures are associated with the outcome? What exposure is most significant? Pattern recognition: Are there specific exposure patterns in the data? Predefined groups: What is the association between outcomes and pre-defined exposure groups? Interactions and non-linearity: Are there interactions between exposures, and if so, which influences modify patterns? Is the exposure-response surface non-linear?
Stafoggia (21) and Yu (22)	Dimensionality reduction; Variable selection; Observational grouping.

The study of multi-pollutant mixtures is characterized by two primary focuses: (A) Estimating the effects of pollutants and (B) addressing the complexity associated with multi-pollutant mixtures.

Regarding effects estimation, the focus is divided into three aspects: (1) Overall effects of multi-pollutant mixtures; (2) Independent effects of components within multi-pollutant mixtures; and (3) Joint effects of mixture components.

Addressing the complexity of multi-pollutant mixtures involves three main aspects: (1) Addressing the challenge of high-dimensional data when multiple chemical substances are present in the model; (2) Resolving the issue of high correlations among pollutants to assess synergistic or antagonistic effects; and (3) Addressing interplay and non-linear effects among pollutants.

Thus, diverse statistical methods are introduced in this paper to address specific issues, as shown in Graphical abstract.

## 2 Methods for effects estimation

**Figure 1:**
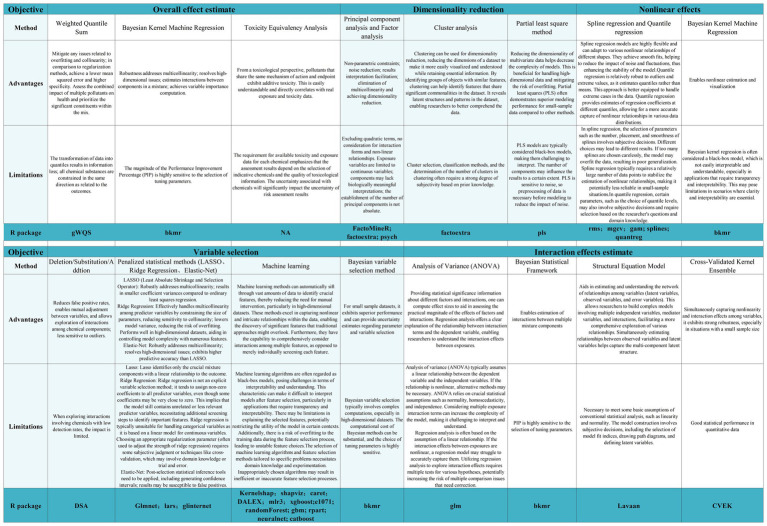
Overview of the methods and R packages for implementation.

In estimating overall effects of multi-pollutant mixtures, two main approaches are commonly used: treating the mixtures as a single exposure or analyzing the weighted sum of exposures (17). For example, particle matter concentration serves as a comprehensive measure of particulate matter components in ambient air, assuming equal impact on health for all components (23). Alternatively, overall effect estimation can involve the weighted sum of individual component effects, with weights based on toxicological potency or contribution percentage (17). For instance, modeling phthalate metabolites’ concentrations using molar sum or potency-weighted sum methods (24, 25). Independent effects estimation considers diverse pollution components’ varied adverse effects, requiring the evaluation of overall mixture impact before assessing individual component effects (26). Joint effects estimation accounts for component interactions beyond additive impacts (18), considering mechanisms and pathways. For example, non-volatile carbonaceous particles like black carbon may require specific attention when studying joint health effects with volatile organic compounds. In this section we briefly introduce several methods of effects estimation. Figure 1 shows details of the effect estimation methods and R packages for their implementation.

### 2.1 Weighted quantile sum regression

Weighted quantile sum (WQS) regression is a convenient tool for addressing effect estimation problem, the high-dimensional and highly correlated issues among multiple pollutants, particularly among homogenous pollutants (27). It is widely used in studies of environmental exposure to multi-pollutant mixtures and enables the identification of high-risk factors. This model allows the construction of a weighted index in a supervised manner to assess the overall effects of environmental exposure and the contribution of each component in the mixture to the overall effect. WQS calculates individual exposure characteristics by weighting based on the correlation between exposure and outcome, resulting in a composite index value for various exposure components. This index characterizes the levels of mixed exposure to a range of exposure components and evaluates the impact of each component on health outcomes. Following Tanner’s recommendation, introducing a bootstrap step in the WQS yields stable weights for exposure components and WQS index estimates (28). The core idea is to construct WQS to achieve dimensionality reduction, address multicollinearity issues, and filter high-risk factors through the weighting process. The most recent weight coefficient for each component in the exposure index represents its contribution to health outcomes.

WQS has advantages in analyzing multifactor exposure due to their simple model structure, small computational burden, and fast analysis speed. But the “directional consistency” precondition must be met, i.e., the effects of each component in the mixture are all in the same direction (all positive or all negative). Recent research has explored and developed new methods for WQS. For unidirectional hypotheses, methods such as quantile g-computation combined with the g-algorithm (29), grouped WQS (30), and Bayes group WQS model (31) have been developed. Focus has also been given to the lagged WQS to address time-varying exposure mixtures (32, 33).

### 2.2 Bayesian kernel machine regression

Bayesian kernel machine regression (BKMR) also provides a new approach to analyze multi-pollutant mixtures (34, 35). In contrast to WQS, BKMR provides probabilities included in the total effects of multi-pollutant mixtures, rather than estimate the percentage contribution of this effect but provides probabilities included in the total effects of multi-pollutant mixtures. It visualizes various exposure-response shapes. BKMR can also examine the independent impacts of mixture components by considering the effects of keeping other components constant at predetermined percentiles, such as the 50th percentile of the exposure distribution. BKMR does not require setting a parameter expression, allowing for the presence of nonlinear effects and interactions. It generates kernel functions based on the mixture variables included in the model, followed by Bayesian sampling and analysis methods to generate relationship curves between mixture components and disease variables included in the model.

In addition to analyzing the mixture’s overall impacts and each component’s effects separately, BKMR also estimates any possible interactions between the distinct components. Posterior inclusion probabilities (PIPs) generated by BKMR range from 0 (least important) to 1 (most important). Components with PIP ≥ 0.5 are identified as relatively important mixture components. BKMR can also be used to study possible three-way interactions. This is achieved by fixing one of the exposures at different quantile levels and visualizing the exposure-response functions for the remaining two exposures. Overall, BKMR has been widely used in environmental health research, including the analysis of continuous variables, binary variables, and repeated measurement data (36, 37). The advantages of this method include the ability to simultaneously assess the importance of each variable, analyze data with uncertainty, and easily extend the obtained results to longitudinal data.

Although BKMR can effectively assess the health effects of multi-pollutant mixtures, it has certain limitations. Firstly, when using the BKMR, the studied exposure variables must be continuous, and the size of PIPs is easily influenced by adjustment parameters. So, caution is required when interpreting results, as this method may obscure the underlying complex features of the data. If some components in the mixture are positively correlated while others are equally negatively correlated, the final overall result will appear as if there is no correlation, and other methods are needed to verify the estimation of their interactions. In addition, considering causality, time-varying exposure, or computational efficiency in massive datasets, the traditional implementation of BKMR may be limited. Several new methods have extended the BKMR strategy to address these limitations, such as bayesian kernel machine regression – causal mediation analysis (BKMR-CMA) (38), bayesian kernel machine regression distributed lag model (BKMR-DLM) (39).

### 2.3 Toxicity equivalency analysis

In addition to the two commonly used estimation methods mentioned above, pollutants with similar mechanisms of action and the same endpoint from a toxicological perspective exhibit additive toxicity. However, individual pollutants contribute differently to the overall health risk. Therefore, a normalization method, known as toxicity equivalency factor (TEF) analysis, a normalizing technique, is required. TEF is generally obtained by comparing the “starting point” of health risk assessments for standard reference compounds with the respective compounds. The exposure dose of a mixture, commonly represented as toxicity equivalent quantity (TEQ), is calculated by multiplying the TEF for each compound by its exposure metric and summing them. By combining TEQ with reference metrics such as the reference dose (RfD) or carcinogenic slope factor, the health risk of the mixture can be assessed (40–43). TEF represents the relative toxicity of an isomer of a compound and is set to 1 for the most toxic 2,3,7,8-TCDD. Other pollutants’ toxicities are converted to their corresponding relative toxic intensities. Alternatively, TEF can be the toxicity equivalency factor for individual Polycyclic Aromatic Hydrocarbons, with a TEF of 0.001 for Pyrene. Daily total intake exposure metric from plasma polycyclic aromatic hydrocarbon levels based on pharmacokinetic models (40–42). The results can be compared against specified standards to determine the presence of carcinogenic risk (43). To address non-linear problems, the acceptable concentration range model has been developed based on the RfD concept (44).

TEF has the advantage of being easy to understand and directly associated with real exposure and toxicity data. However, it requires available toxicity and exposure data for each chemical, making the assessment results dependent on the selection of indicative chemicals and the quality of toxicological information. Uncertainty in the chemicals significantly affects the uncertainty of risk assessment results.

### 2.4 Other methods for effects estimation

In addition to the three statistical methods for estimating effects mentioned above, there are also novel and unique methods for effect estimation, although they may have a narrower audience. These include bayesian regression trees (45), bayesian data synthesis (BDS) (46), bayesian subset selection and variable importance for interpretable prediction and classification (BSSVI) (47), directed acyclic graph analysis (48), bayesian treed distributed lag model (DLMtree) (49), factor analysis for interactions (FIN) (50), parametric decision analysis method (51), graph laplacian-based gaussian Process (GL-GPs) (52), computational improvements for bayesian multivariate regression models based on latent meshed gaussian processes (GriPS) (53), and multiple exposure distributed lag model with variable selection (54).

## 3 Methods for dimensionality reduction

When analyzing multi-pollutant mixtures with fewer components, the process is relatively simple, but the dimensionality of the data increases dramatically when multi-pollutant mixtures contain several components. Many statistical methods lack the capability to address this issue, and even methods designed to handle the complexity of high-dimensional data incur exponential time costs as the data dimensionality grows. Furthermore, the high correlation among components may lead to multicollinearity. For instance, analyzing correlated components with similar sources, exposure pathways, or metabolic processes, regardless of which one is individually studied, may yield biased conclusions. Faced with the challenges of high dimensionality and multicollinearity, a crucial aspect of studying the health impacts of multi-pollutant mixtures involves learning low-dimensional structures in the data to enhance interpretability and statistical efficiency, employing methods of dimensionality reduction proves to be a favorable approach. In this section we briefly introduce several dimensionality reduction methods. Figure 1 shows details of the methods for dimensionality reduction and R packages for their implementation.

### 3.1 Principal component analysis and factor analysis

Principal component analysis (PCA), introduced by Pearson for non-random variables and later extended to random vectors by Hotelling, transforms a set of potentially correlated variables into a set of linearly uncorrelated variables, referred to as principal components, through orthogonal transformation (55). The primary objective of PCA is to explain the majority of variance in the original data using fewer variables, converting highly correlated variables into ones that are independent or uncorrelated. When analyzing the relationship between multiple pollutant indicators and health, PCA can reduce the number of indicators for analysis, minimizing information loss from the original indicators and facilitating comprehensive data analysis. It simplifies high-dimensional exposure data into several orthogonal components usable for regression models, thus mitigating multicollinearity issues. For example, Smit applied PCA to estimate the relationship between the risk of asthma and eczema in school-age children and 16 pollutants in their mothers’ serum. The study ultimately incorporated indicators from five principal components, explaining 70% of the variance in the outcome variable (56).

PCA’s main limitations include difficulty in interpreting results, as the components are not in the same units as the original exposure variables, and the derived components may lack a direct relationship with study outcomes as they are derived in an unsupervised manner. Subsequently, PCA has evolved into methods like supervised PCA, which overcomes these issues by excluding “pollutants” that do not provide information directly related to the outcomes (57). Roberts applied this method to air pollution analysis, proposing a recursive algorithm that identifies the optimal predictor for study outcomes and combines it into several relevant principal components (58). Other developments include principal component pursuit (PCP), an analysis method based on matrix factorization, extended to multi-pollutant mixtures by Gibson. Through cross-validation in simulations, PCP identified the true number of patterns in all simulations, while PCA achieved this in only 32% of simulations, demonstrating PCP’s superiority in most simulation scenarios (59). In addition to the above methods, Positive matrix factorization (PMF) is a variant of PCA applicable to multi-pollutant profiles, deriving air pollution sources from individual chemical components (60). Specifically, PMF decomposes the matrix of mixture data into two matrices—source contributions and source profiles. Source contributions represent the mass contribution of each source to the mixture measurements, while source profiles reflect the emission types from a given source. Source contributions are constrained to be non-negative, and the method can incorporate uncertainty measurements related to the data at each point (61–64).

Factor analysis (FA) is another commonly used dimensionality reduction method that groups variables based on the correlation matrix, creating common factors that represent the fundamental structure of the data. It decomposes multidimensional variables into a small number of common factors, where the fundamental idea is to break down original variables into two parts: one part is a linear combination of common factors that condenses a vast majority of information in the original variables, and the other part is special factors unrelated to common factors, reflecting the gap between the linear combination of common factors and the original variables. In other words, FA aggregates numerous variables into a few independent common factors with minimal loss or little loss of original data information. These common factors can reflect the essential information of numerous variables, reduce the number of variables, and reveal the inherent connections among variables. Perturbation FA is commonly used in multi-pollutant mixtures studies, focusing on exploring the similarities and differences in exposure conditions among different groups. For example, Roy used this method to assessed the differences in exposure characteristics in biological or social structures based on race/ethnicity (65).

Both PCA and FA seek a small number of variables to comprehensively reflect the majority of information in all variables. While the number of variables is fewer than the original variables, the information contained is substantial, and the reliability of using these new variables for analysis remains high. Moreover, these new variables are uncorrelated, eliminating multicollinearity and achieving dimensionality reduction. In PCA, the newly determined variables are linear combinations of the original variables, obtained through coordinate transformation. In contrast, FA aims to explain the complex relationships present in many observed variables using a small number of common factors. It does not recombine original variables but decomposes them.

### 3.2 Clustering analysis

Clustering analysis (CA) organizes all data into clusters, groups of similar elements, where instances within the same cluster are similar to each other, while those in different clusters are dissimilar. Similarity among data is determined by defining a distance or similarity coefficient (66). Once several clusters are identified, the next step is to select a representative prototype for each cluster. CA can be matched with exposure data to define groups, and indicators of group members can then be used as predictor variables in health outcome regression models.

Clustering can be categorized into different groups based on techniques, with partition-based clustering being the most widely used, where k-means is a common approach (67). One advantage of the k-means method is its linear complexity, making its execution time proportional to the number of individuals, making it suitable for large datasets. However, the choice of initial centers and the number of clusters is arbitrary and can influence the results. Nevertheless, hierarchical classification can be applied to the cluster centers obtained from the k-means method. Clustering has been used in several studies to assess the impact of various pollutants. For instance, in time series analysis of air pollution, one study used k-means to divide days into five groups representing days with low pollution levels, high concentrations of crustal particles, high particle content from traffic and combustion of oil, days influenced by regional pollution sources, and days with high concentrations of particles from wood or oil burning (68). Some clusters were associated with pulse amplitude. Similarly, based on pollutant characteristics and community background, an evaluation was conducted on the correlation between NO_2_, NO, and PM_2.5_ concentrations and low birth weight (69).

The challenges of CA lie in the selection of clusters, classification methods, and the number of clusters. CA facilitates the distinct grouping of various entities, making it challenging to summarize them under a single label. The process typically necessitates the initial selection of appropriate distance metrics, clustering algorithms, and the number of clusters. These choices often based on users’ subjective judgments by the user, and different selections may yield disparate clustering outcomes, thereby rendering the results subjective.

## 4 Methods for variable selection

When analyzing multi-pollutant mixtures with many components, it is not necessary to estimate the impact of each component of mixture, rather, the focus is on investigating the effects of a few crucial components that exhibit the maximum toxicity to human health and/or have the highest predictive power for the outcomes of interest. Therefore, it is imperative to employ appropriate methods for identifying or selecting important variables that represent the exposure-response relationship between individual exposures in the mixture and the outcomes. Uch methods are frequently known as “variable selection.” In this section we briefly introduce several methods of variable selection. Figure 1 shows details of the methods for variable selection and R packages for their implementation.

### 4.1 Partial least squares

Partial least squares (PLS) regression combines principal component analysis and multivariate regression, taking into account the correlation between the outcomes and exposure variables (70). In essence, PLS regression searches for a linear decomposition of the exposure matrix that maximizes the covariance between exposure and outcomes. The exposed variable has a higher weight in the linear combination, the stronger the association between it and the outcome. PLS regression can also include multiple outcome variables. The optimal number of components can be selected based on cross-validated mean squared error (71). However, a drawback of PLS regression is that the interpretation of the linear combination can be challenging, especially in the presence of a large number of original exposure variables.

To address this limitation, Chun and Keles introduced a method called sparse PLS regression, which simultaneously combines variable selection and dimensionality reduction (72). This method results in a linear combination of exposure variables with reduced quantity. Sparsity is introduced into the loadings of exposure variables through penalty terms. The optimal number of components and sparsity parameters are selected based on cross-validated performance. This method has been applied in simulation studies related to exposure-health associations. In one simulation study involving 237 generated exposure covariates, 0 to 25 of which were related to the outcomes, sparse PLS regression demonstrated better sensitivity in distinguishing true predictive factors from correlated covariates (73).

### 4.2 Deletion/substitution/addition algorithm

The Deletion/Substitution/Addition (DSA) algorithm is a variable selection method (74, 75). The main steps include: (1) removal of selected variables; (2) substitution of selected variables with unselected ones; and (3) addition of new variables. By using five-fold cross-validation to minimize the root mean square error (L2 loss function) of the prediction equation, the number and particular kinds of variables included in the model are ascertained. To ensure selection stability, DSA is run with different seed numbers for 50 iterations. Subsequently, a binomial generalized linear model evaluation is conducted for multi-exposure variables. Variables included in the final model are those selected in at least 6% (*n* ≥ 3 times) or 10% (*n* ≥ 5 times) of DSA iterations, and multicollinearity is validated in the final model.

Compared to traditional linear regression equations, this method reduces the false-positive rate, allows for mutual adjustments between variables, and explores interactions between chemicals. However, its effectiveness is limited when exploring interactions involving chemicals with low detection rates. The algorithm also provides the possibility of including interaction terms. In contrast to stepwise model selection procedures, DSA has the advantage of being less sensitive to outliers and permits movement between non-nested statistical models. In previous applications, the DSA algorithm has been utilized in multi-pollutant mixtures analysis, estimating the relationship between O_3_, CO, NO_2_, PM_10_ and lung function (76). However, DSA has faced criticism, particularly when the ratio of sample size to the number of candidate predictors is small, leading to inconsistent estimates. Moreover, its statistical properties for confidence intervals are compromised, when there is substantial correlation between predictors (77).

### 4.3 Penalty-based algorithms

Least absolute shrinkage and selection operator (LASSO) regression is highly similar to ordinary least squares, with the key difference lying in the estimation of coefficients through the minimization of a slightly different quantity, resulting in a shrinkage penalty on the coefficients’ magnitudes (78). It penalizes the absolute size of regression coefficients based on the value of the tuning parameter λ. Consequently, LASSO can drive coefficients of irrelevant variables to zero, thereby performing automatic variable selection. When the tuning parameter λ is small, the results essentially converge to least squares estimation. Elastic net (ENET) combines the LASSO method with ridge regression (RR) (79)，it includes first and second-order penalty terms on the regression coefficients. Thus, it not only selects the best subset of variables by precisely shrinking some effect estimates to zero through LASSO but also retains a set of highly correlated variables in a RR model with similar effect estimates. For instance, in the Veterans Affairs Normative Aging Study, the use of LASSO enables the selection of PM_2.5_ components related to blood pressure (80). In a recent study, based on ENET penalized regression, two metabolites of phthalates were found to be consistently associated with impaired fetal growth (81). The group-lasso interaction-net method extends LASSO to select bidirectional interaction terms (82), allowing for the simultaneous use of LASSO while controlling the false discovery rate (83). A key characteristic of LASSO is the introduction of an L1 regularization term in estimation, leading to the precise compression of certain coefficients to zero, thereby achieving feature selection. Nevertheless, this excessive sparsity may render the model overly sensitive to noise, and the selected features may prove unstable across different datasets.

### 4.4 Machine learning approaches

Machine learning (ML) is a research methodology focused on discovering patterns within data and utilizing these patterns to make predictions. Variable selection is a crucial issue in the field of ML, as the predictive performance of models is influenced to some extent by the variables included in the model. The number of variables, variables’ correlations, and the inclusion of important variables significantly impact the accuracy and efficiency of predictive models. Therefore, variable selection plays an indispensable role in constructing predictive models. Numerous ML algorithms are currently available for variable selection based on variable importance. Common methods include classification and regression trees (84), random forest (RF) models (85), support vector regression (SVM) (86), K-nearest neighbors (KNN) (87), naive bayes (88), neural networks (89), adaptive boosting (AdaBoost) (90), gradient boosting (GBM) (91), eXtreme gradient boosting (XGBoost) (92), light gradient boosting machine (LightGBM) (93), CatBoost (94), and others are examples of common techniques.

While ML demonstrates effective results, they often face challenges related to interpretability. For instance, models like XGBoost or LightGBM, comprised of *N* trees, make it difficult to understand how the features of a specific sample influence the final result. To address this issue, SHAP (shapley additive explanations) provides a method for explaining ML, offering detailed and interpretable information about model predictions (95). As the demand for incorporating complex high-dimensional data in environmental health research continues to grow, researchers are increasingly turning to ML. Recent studies have employed various ML such as AdaBoost, SVM, RF, decision tree classifier (DT), and KNN to identify the relationship between heavy metal exposure and coronary heart disease. Integrated with SHAP, these studies explained ML, determining the contributions of heavy metals such as cesium, thallium, antimony, dimethyl arsenic acid, barium, and arsenic acid in urine to the risk of coronary heart disease. This increases the likelihood that coronary heart disease can be detected and treated early (96). Some studies have also used multilayer perceptron, RR, gradient boosting decision tree, voting classifier, and KNN algorithms for generating optimal predictive models for multiple heavy metals causing hypertension. These studies integrated permutation feature importance analysis, Partial Dependence Plots, and SHAP methods into a single process, embedded within ML for model interpretation (97). However, most of the mentioned ML models are used for prediction and require comparisons based on accuracy, sensitivity/recall, specificity, negative predictive value, false positive rate, false negative rate, and F1 score.

### 4.5 Bayesian variable selection methods

ML algorithms such as RF can provide measures of variable importance for mixed components, but these measures do not succinctly capture the overall magnitude or direction of their associations. Variable selection techniques within the regression framework, such as LASSO, shrink individual regression coefficients to zero. However, these techniques are typically based on relatively simple models of mixed components parameters. To systematically address highly correlated exposures, the BKMR employs a hierarchical variable selection approach. This method can incorporate prior knowledge about the exposure variable/mixed component correlation structure to provide PIPs, as detailed in Section 2.2.

## 5 Methods for identifying multi-exposure interactions

Although various components in multi-pollutant mixtures may have completely independent effects on health outcomes, in many cases, there may be interactions among components in the mixtures. Interactions represent the mutual dependence effects of two or more variables and can manifest as synergistic, additive, or antagonistic effects (98). A typical example of interaction is the additive synergistic effect of O_3_ and particulate matter on the incidence of cardiovascular diseases (99). In the real world, interactions among various exposure pollutants may exist, and the analysis of these interactions aims to identify and explain their effects. Analyzing and interpreting interactions among multiple exposures can provide a more comprehensive understanding of exposure patterns and identify cooperative effects between specific exposures under certain conditions. In this section we briefly introduce several methods for identifying interaction effects. Figure 1 shows details of the methods for identifying multi-exposure interactions and R packages for their implementation.

### 5.1 Basic interaction analysis

In interaction factor analysis, analysis of variance (ANOVA) is commonly used to test whether interaction effects among multiple exposures are significant. By comparing the *F*-values or *p*-values of individual factors and interactions, it is possible to determine which factors exhibit interaction effects. Additionally, regression analysis can also be used to explore the interaction effects of exposures, perform significance tests, and create a relationship model between exposure interaction terms and outcomes.

### 5.2 Bayesian statistical framework

Apart from BKMR, Antonelli utilized a semi-parametric Bayesian sparse prior regression framework to generate variable importance scores for each exposure and each pairwise interaction in the mixture (100).

### 5.3 Structural equation model

A technique called the structural equation model (SEM) combines particular covariance and regression sets between certain variables into a single coherent model (101). It is used to test and estimate relationships between observed data and latent variables, as well as to assess the fit of theoretical models. SEM combines various techniques such as FA and path analysis, allowing researchers to simultaneously explore complex relationships between multiple variables. In SEM, a measurement model can be constructed to capture measurement errors and covariances among different exposure factors and health outcomes. This aids in accurately measuring these factors and accounting for measurement errors. It is also possible to determine the causal links between various exposure factors and health outcomes using structural models.

SEM is useful for estimating and understanding the network of relationships between variables (latent, observed, and error variables) and it is also employed to estimate the degree of model fit and allows for the presence of measurement errors in independent and dependent variables (102). As researchers turn to modeling multi-pollutant mixtures, SEM is increasingly used to estimate the impact of multi-pollutant mixtures on health (103, 104). For instance, SEM assessed the relationship between respiratory function, tobacco smoke exposure, and volatile organic compound exposure in a nationally representative sample of adolescents, revealing associations between respiratory function and certain types of volatile organic compounds (104). It is noteworthy that a critical feature of SEM analysis is its requirement to meet some basic assumptions of traditional statistical analyses, such as linearity and normality; otherwise, the obtained statistical data may be unreliable.

## 6 Methods for nonlinear effects

Numerous epidemiological studies have identified nonlinear associations (U-shaped, inverted U-shaped, J-shaped, etc.) between mixed pollutant exposures and health outcomes. For example, the relationship between plasma heavy metals concentrations and type 2 diabetes (105), as well as the association between volatile organic compounds and heart rate variability index (106). Ignoring the potential nonlinearity may result in biased conclusions. Therefore, a better approach is to fit the nonlinear relationship between exposure and outcome. In this section we briefly introduce several methods for estimating nonlinear effects. Figure 1 shows details of the methods for nonlinear effects and R packages for their implementation.

### 6.1 Spline regression and quantile regression

To overcome the limitations of polynomials, spline methods are often used for curve fitting, employing a piecewise function strategy instead of complex polynomials. One commonly used method in pollution studies is restricted cubic spline (RCS), which fits the curve relationship between a variable and an outcome using restricted cubic spline terms. For instance, Zhou combined RCS with logistic regression to estimate the relationship between typical heavy metal contents (lead, cadmium, mercury, and manganese) in the blood of adults and the metabolic syndrome (107). Similarly, generalized additive models can fit spline line models without specifying nodes automatically, allowing the fitting of spline terms like B-splines, natural splines, thin plates, etc., to control the impact of nonlinear confounding factors. This is achieved by fitting curves of corresponding nonlinear terms of pollutants.

Quantile regression (QR) is a regression analysis method that allows modeling different quantiles of the dependent variable, it can handle issues like non-normal error distribution, heteroscedasticity, and outliers. QR can also be used to fit the nonlinear relationship between pollutants and outcomes. For example, a study used linear regression and QR to investigate the relationship between the increase in concentrations of pollutants (PM_10_, PM_2.5_, NO_2_, and O_3_) and changes in birth weight by using linear regression and QR (108).

### 6.2 Bayesian kernel machine regression

BKMR can also handle nonlinear relationships between exposures. Exposure and result interactions are frequently nonlinear, and BKMR is an efficient way to capture these kinds of nonlinear relationships between contaminants. BKMR has been applied to a dataset on metal exposure and neurodevelopment in Bangladeshi children, indicating the presence of non-additive and nonlinear exposure-response functions between metals and a summary measure of psychomotor development (109).

### 6.3 Other methods for nonlinear effects

Methods highlighted in other categories can also be employed to address nonlinear problems, including cross-validated ensemble of kernels (110), TEV, BSSVI, BVSM, MatchAlign, BDS, BKMR-CMA, GriPS, SGP-MPI, BMIM, GL-GPs, Bayesian Tree Ensembles, BKMR-DLM, DLMtree, SPORM, FIN, and FOTP.

## 7 Holistic approaches to mixture studies

As understanding of environmental pollution deepens and technology advances, the study of a single pollutant can no longer match the analysis of the total health impact of pollutants on the human body. So, researchers are increasingly recognizing the need to analyze complex interactions leading to or exacerbating diseases in mixtures. It is imperative to evaluate the connections between various risk factors and modifying factors from multiple biological dimensions. Similar to exploring the impact of genetic factors on chronic diseases through genome-wide association studies, exposome-wide association studies (EWAS) facilitate the investigation of non-genetic risk factors.

Initially proposed by Wild (111), EWAS can be represented as P = G + E, where an individual’s phenotype, encompassing health and physical characteristics, is the sum of genetic factors (G) and environmental factors (E) (112). Rappaport also argue that exposure should not be limited to directly encountered chemicals but should consider a broader range of exposures, such as microbial exposure and life stress (113). EWAS provides a conceptual framework to understand the complex network of interactions between genes and the environment, as well as their causal relationships with diseases. It facilitates a holistic analysis of the impact of genetics and the environment on human diseases, including DNA sequences, epigenetic DNA modifications, gene expression, metabolite analysis, and the intricate and dynamic interactions among environmental factors, all of which can influence disease phenotypes.

EWAS research does not solely focus on a single exposure but systematically addresses multiple exposures and their mutual influences, thereby increasing the complexity of the study (114, 115). For instance, a recent study utilized data from the National Health and Nutrition Examination Survey (NHANES) and retained exposure factors, including 75 laboratory variables (clinical and biological biomarkers of environmental chemical exposure) and 64 lifestyle variables (63 dietary variables and 1 physical exercise variable). This study described the associations between body mass index, nutrition, clinical factors, and environmental factors among adolescents (116).

## 8 Conclusion

In conclusion, the statistical analysis of health effects resulting from the multi-pollutant mixtures is a key challenge in current environmental epidemiological research. In this paper, we review multi-pollutant mixtures statistical methods. It is essential to note that while examining scientific ideas, complementary approaches should be taken into account and statistical methods should be selected with the particular scientific problems in mind. By selecting appropriate statistical methods, considering the combined effects of various pollutants, incorporating interdisciplinary collaboration and emerging technological tools, a more accurate and comprehensive assessment of the impact of mixed environmental pollutant exposure on human health can be achieved. This will contribute to the scientific basis for environmental protection and the formulation of public health policies, promoting sustainable development for human health.

To facilitate the application of the discussed statistical methods, we summarize the advantages and limitations of commonly used statistical methods, corresponding R packages, and the above basic statistical analyses were conducted using the NHANES dataset within gWQS package (Please refer to Figure 1; Supplementary material for statistical analysis code). This serves as a convenient resource for researchers to directly apply these methods.

## Author contributions

GZ: Writing – review & editing, Visualization, Writing – original draft. YW: Visualization, Writing – original draft, Writing – review & editing. KC: Visualization, Writing – review & editing. SH: Visualization, Writing – review & editing. TW: Writing – review & editing, Funding acquisition, Supervision.
