# Supplementary material for: A review of common statistical methods for dealing with multiple pollutant mixtures and multiple exposures
Source: Front Public Health. 2024 May 9;12:1377685. doi: 10.3389/fpubh.2024.1377685 (PMC11113012; doi:10.3389/fpubh.2024.1377685)
Supplement: Supplementary file 1 [file Table_1.docx]

Contents

[1. Methods for Effects Estimation 1](#_Toc163244263)

[1.1 Weighted Quantile Sum(WQS) 1](#_Toc163244264)

[1.2 Bayesian Kernel Machine Regression(BKMR) 3](#_Toc163244265)

[2. Methods for Dimensionality Reduction 5](#_Toc163244266)

[2.1 Principal Component Analysis(PCA) 5](#_Toc163244267)

[2.2 Factor Analysis(FA) 6](#_Toc163244268)

[2.3 Cluster Analysis(CA) 7](#_Toc163244269)

[3. Methods for Variable Selection 8](#_Toc163244270)

[3.1 Partial Least Squares(PLS) 8](#_Toc163244271)

[3.2 Penalty-Based Algorithms (LASSO/Ridge Regression/Elastic-Net) and Least absolute shrinkage and selection operator (LASSO) 9](#_Toc163244272)

[3.3 Ridge Regression(RR) 10](#_Toc163244273)

[3.4 Elastic-Net(ENET) 11](#_Toc163244274)

[3.5 Machine Learning(ML) Approaches 12](#_Toc163244275)

[3.5.1 Random Forest(RF) 12](#_Toc163244276)

[3.5.2 Support Vector Machine(SVM) 13](#_Toc163244277)

[3.5.3 Decision Tree(DT) 14](#_Toc163244278)

[3.5.4 Gradient Boosting Tree Model 15](#_Toc163244279)

[3.6 Other machine learning algorithm variable screening 16](#_Toc163244280)

[3.6.1 Variable screening of CARET random forest 16](#_Toc163244281)

[3.6.2 mlr3 package random forest variable screening 17](#_Toc163244282)

[3.6.3 Bayesian variable selection method 18](#_Toc163244283)

[4. Methods for Identifying Multi-Exposure Interactions 18](#_Toc163244284)

[4.1 Single factor analysis and linear model with interaction terms 18](#_Toc163244285)

[4.2 Two-Way ANOVA sample code 18](#_Toc163244286)

[4.3 Bayesian Kernel Machine Regression(BKMR) 19](#_Toc163244287)

[5. Methods for Nonlinear Effects 20](#_Toc163244288)

[5.1 Restricted Cubic Spline(RCS) 20](#_Toc163244289)

[5.2 Generalized Additive Model (GAM) 20](#_Toc163244290)

[5.3 Quantile Regression (QR) 21](#_Toc163244291)

[5.4 Bayesian Kernel Machine Regression(BKMR) 21](#_Toc163244292)

[5.5 Cross-Validated Kernel Ensemble (CVEK) 22](#_Toc163244293)

# 1. Methods for Effects Estimation

## 1.1 Weighted Quantile Sum(WQS)

#Install and load the R package

install.packages("gWQS")

install.packages("epiDisplay")

install.packages("ggplot2")

library(gWQS)

library(epiDisplay)

library(ggplot2)

#Import Data

data(wqs_data)

#View data variables and types

str(wqs_data)

toxic_chems <- names(wqs_data)[1:34]

wqsfit<- gwqs(yLBX ~ wqs + sex, mix_name = toxic_chems, data = wqs_data, q = 4, validation = 0.6, b = 1000, b1_pos = TRUE, b1_constr = FALSE, family = "gaussian", seed = 2023, plots = TRUE, tables = TRUE)

#Conduct gWQS (generalized weighted quantile sum regression), where the relationship between the dependent variable y and the estimated WQS index based on quartile exposure concentration ranking (q=4);

#b=1000 is the number of bootstraps;

#60% of the samples are used as validation set;

#You can also set the b1_pos parameter to false (b1_pos=false) to test the negative association

#seed is the seed point

#family is the link function

#View results

summary(wqsfit)

#View overall trends

gwqs_scatterplot(wqsfit)

#residual graph

gwqs_fitted_vs_resid(wqsfit)

#Detailed model information

ptbp<-wqsfit$final_weights

ptbp

round(ptbp$mean_weight,4)

gwqs_summary_tab(wqsfit)

ptb<-regress.display(wqsfit$fit)

ptb

#View the weight of each factor

gwqs_barplot(wqsfit)

#View the weight of each factor and optimize visualization

weight = as.data.frame(wqsfit$final_weights$mean_weight)

label = as.data.frame(wqsfit$final_weights$mix_name)

final_weight = cbind(label,weight)

View(final_weight)

w_ord <- order(wqsfit$final_weights$mean_weight)

mean_weight <- wqsfit$final_weights$mean_weight[w_ord]

mix_name <- factor(wqsfit$final_weights$mix_name[w_ord],

levels = wqsfit$final_weights$mix_name[w_ord])

data_plot <- data.frame(mean_weight, mix_name)

ggplot(data_plot, aes(x = mix_name, y = mean_weight, fill = mix_name)) +

geom_bar(stat = "identity", color = "black") + theme_bw() +

theme(axis.ticks = element_blank(),

axis.title = element_blank(),

axis.text.x = element_text(color='black'),

legend.position = "none") + coord_flip()

## 1.2 Bayesian Kernel Machine Regression(BKMR)

#Install and load the R package

install.packages("bkmr")

install.packages("ggplot2")

library(bkmr)

library(ggplot2)

#Set exposed variables

covar <- data.matrix(as.numeric(wqs_data[, c("sex")]))

expos <- data.matrix(wqs_data[1:34])

y<-wqs_data$yLBX

#Similarly set the number of seeds

set.seed(123)

# perform analysis

fitkm <- kmbayes(y = y, Z = expos , X = covar, iter = 10000, verbose = FALSE, varsel = TRUE)

ExtractPIPs(fitkm)

#Single variable mode

pred.resp.univar <- PredictorResponseUnivar(fit=fitkm, q.fixed = 0.5)

ggplot(pred.resp.univar, aes(z, est, ymin = est - 1.96*se, ymax = est + 1.96*se)) +

geom_smooth(stat = "identity") + facet_wrap(~ variable) + ylab("h(z)")

#Multivariable interaction analysis

pred.resp.bivar <- PredictorResponseBivar(fit = fitkm, min.plot.dist = 1)

#Analysis of the overall position at a specific percentile

risks.overall <- OverallRiskSummaries(fit = fitkm, qs = seq(0.1, 0.9, by = 0.05), q.fixed = 0.5)

# 2. Methods for Dimensionality Reduction

## 2.1 Principal Component Analysis(PCA)

#Install and load the R package

install.packages("FactoMineR")

install.packages("factoextra")

install.packages("psych")

library(FactoMineR)

library(factoextra)

library(psych)

# Analyze exposed data

expos <- wqs_data[1:34]

# Principal component analysis PCA

if (T) {expos.pr<-princomp(expos,cor=TRUE)

summary(expos.pr,loadings=TRUE)

}

#The second step is to determine the number of principal components

fa.parallel(expos , fa = "pc", n.iter = 100,

show.legend = F, main = "Scree plot with parallel analysis")

#Use 17 factors for regression analysis

pre<-predict(expos.pr)

str(pre)

Z<-pre[,1:17]

head(Z)

str(Z)

pcadata<- data.frame(Z,wqs_data$yLBX)

str(pcadata)

lmfit<-lm(wqs_data.yLBX~.,pcadata)

summary(lmfit)

## 2.2 Factor Analysis(FA)

#Install and load the R package

install.packages("psych")

library(psych)

#The second step is to determine the number of factors

fa.parallel(expos , fa = "fa",

show.legend = F, main = "Scree plot with parallel analysis")

# Carry out factor analysis. First, use the 17 factors to see the results. Maximum likelihood method, no rotation.

fa.res <- fa(expos, nfactors = 17, rotate = "none", fm="ml")

fa.res

# Select 4 factors, use maximum variance rotation, maximum likelihood method

fa.res <- fa(expos, nfactors = 17, rotate = "varimax", fm="ml")

fa.res

fa.diagram(fa.res)

factor.plot(fa.res,labels=rownames(fa.res$loadings))

fa.diagram(fa.res,simple=F,rsize=1.5,adj=2)

#Residual same principal component analysis

## 2.3 Cluster Analysis(CA)

#When performing K-means clustering, you need to specify the number of clusters at the beginning

#Install and load the R package

install.packages("factoextra")

library(factoextra)

set.seed(123)

fviz_nbclust(expos, kmeans, k.max = 10)

#Perform K-means clustering, select 3 centers, centers = 3.

set.seed(123)

fit.km <- kmeans(expos, centers = 3, nstart = 25)

fit.km

fviz_cluster(fit.km, data =expos )

#K-means clustering is based on the mean, so it is very sensitive to outliers.

#A more robust approach is division around a center point.

#Use the most representative observation value to represent this category (somewhat similar to a principal component).

#K-means clustering generally uses Euclidean distance and can be calculated using any distance.library(cluster)

set.seed(123)

fit.pam <- pam(expos, k=3, #Group into 3 categories

stand = T # Standardize before clustering

)

fit.pam

fviz_cluster(fit.pam,

ellipse = T, # Add ellipse

ellipse.type = "t", # Ellipse type

geom = "point", # Only display points without text

palette = "aaas", # supports many color schemes

ggtheme = theme_bw() # Support changing themes

)

# 3. Methods for Variable Selection

## 3.1 Partial Least Squares(PLS)

#Install and load the R package

install.packages("pls")

library(pls)

plsdata<-data.frame(wqs_data[,1:34],wqs_data$yLBX)

str(plsdata)

#For example, set the component score 4 here (the first 4 main components are displayed in the results), and perform cross-validation.

plsfit<- plsr(wqs_data.yLBX~., data =plsdata, scale = TRUE, ncomp = 4, validation = 'CV')

summary(plsfit)

#Look at the importance of ingredients and determine which ingredients are appropriate to retain

validationplot(plsfit, val.type = 'MSEP')

validationplot(plsfit, val.type = "R2")

#Since the variables have been standardized by the scale parameter during PLS regression

#Therefore, the absolute value of the regression coefficient can be used as the basis for evaluating the relative importance of variables.

coefficients <- coef(plsfit)

coef_weight <- abs(coefficients) #The absolute value of the regression coefficient

coef_sum <- sum(coef_weight)

importance <- coef_weight * 100 / coef_sum #The percentage importance of each variable calculated based on the absolute value of the regression coefficient

importance <- sort(importance[ ,1,1], decreasing = TRUE) #Sort by importance

importance <- cbind(importance, coefficients = coefficients[ ,1,1][names(importance)])

write.table(importance, 'NIR_importance.txt', col.names = NA, sep = '\t', quote = FALSE)

#View the top 10 important variablescoef_top10 <- head(importance, 10)

coef_top10

par(las = 1)

barplot(coef_top10[ ,1], horiz = TRUE, width = 0.6, xlab = 'importance (%)',

col = ifelse(coef_top10[ ,2] > 0, 'red', 'blue'))

## 3.2 Penalty-Based Algorithms (LASSO/Ridge Regression/Elastic-Net) and Least absolute shrinkage and selection operator (LASSO)

#Install and load the R package

#install.packages("glmnet")

library(glmnet)

str(wqs_data)

X <- as.matrix(wqs_data[,1:34])

#The independent variable is columns 1-34

Y <- as.matrix(wqs_data[,35])

# when lambda = 0.01

lambda <- 0.01

#lasso

lafit <- glmnet(X, Y, lambda=lambda,

family='gaussian',

intercept = F, alpha=1)

# When alpha is set to 0, it is ridge regression, and when alpha is set between 0 and 1, it is elastic net.

# Coefficient result (lambda=0.01)

lafit$beta[,1]

lafit

par(mfrow = c(1, 1))

plot(lafit,xvar = "lambda")

mod_cv <- cv.glmnet(x=X, y=Y, family="gaussian", # Default nfolds = 10

intercept = F, alpha=1)

plot(mod_cv)

best_lambda <- mod_cv$lambda.min

best_lambda

# Coefficient estimates of the final model

#find coefficients of best model

best_model <- glmnet(X, Y, alpha = 1, lambda = best_lambda)

coef(best_model)

## 3.3 Ridge Regression(RR)

ridge <- glmnet(X, Y, lambda=lambda,

family='gaussian',

intercept = F, alpha=0)

ridge$beta[,1]

ridge

plot(ridge,xvar = "lambda")

ridgeCV <- cv.glmnet(X, Y, family="gaussian",

alpha=0,intercept = F)

plot(ridgeCV)

best_lambda <- ridgeCV$lambda.min

best_lambda

best_model <- glmnet(X, Y, alpha = 0, lambda = best_lambda)

coef(best_model)

## 3.4 Elastic-Net(ENET)

elastic <- glmnet(X, Y, family="gaussian", lambda=lambda,alpha=0.5)

elastic$beta[,1]

elastic

plot(elastic, xvar="lambda")

elasticCV <- cv.glmnet(X, Y, family="gaussian",

intercept = F,

alpha=0.5)

plot(elasticCV)

best_lambda <- elasticCV$lambda.min

best_lambda

best_model <- glmnet(X, Y, alpha = 0.5, lambda = best_lambda)

coef(best_model)

## 3.5 Machine Learning(ML) Approaches

#Because there are many machine learning algorithms, here we only show a few commonly used methods, such as

### 3.5.1 Random Forest(RF)

#Install and load the R package

install.packages("randomForest")

library(randomForest)

#Random forest calculation (default generates 500 decision trees)

rfdata<- data.frame(wqs_data[,1:34],wqs_data$yLBX)

str(rfdata)

set.seed(123)

forestfit <- randomForest(wqs_data.yLBX~., data = rfdata, importance = TRUE)

forestfit

# Draw the relationship between error and number of trees

plot(forestfit)

# Find the number of trees corresponding to the minimum error of the total sample

which.min(forestfit$err.rate[,1])

# Refit the model, set ntree = 47, mtry sets the number of iterations for each time

set.seed(234) # Because the method has random selection, random numbers need to be set to ensure the reproducibility of the code.

bestforestfit<- randomForest(wqs_data.yLBX~., data = rfdata, ntree = 11, mtry = 11)

bestforestfit

importance(bestforestfit)

varImpPlot(bestforestfit,main="Variable Impprtance Plot")

partialPlot(bestforestfit, pred.data=rfdata, x.var=LBX074LA)

#shap

#Install and load the R package

install.packages("kernelshap")

install.packages("shapviz")

library(kernelshap)

library(shapviz)

#Calculate overall shap

s <- kernelshap(bestforestfit, rfdata[-35], bg_X = rfdata)

sv <- shapviz(s)

#Show overall shap

sv_importance(sv, kind = "both")

#Show the shap of individual 1

sv_waterfall(sv,row_id =1,max_display =2)

### 3.5.2 Support Vector Machine(SVM)

#Install and load the R package

install.packages("e1071")

library(e1071)

#Variable filtering of support vector machines

set.seed(234)

#Set filtering method

install.packages("caret")

library(caret)

selecMeth <- rfeControl(method = 'cv', # Specify cross validation

number = 10) #Specify nfold number

# There are three variable filtering methods in the rfe function: svmLinner; svmPoly; svmRadial

# size specifies the number of independent variables

#Specify independent variables and dependent variables

X <- wqs_data[,1:34]

Y <- wqs_data[,35]

svm.feature <- rfe(X,Y,sizes = c(1:34), rfeControl = selecMeth, method = 'svmRadial')

svm.feature

#shap

svmdata<- data.frame(wqs_data[,1:34],wqs_data$yLBX)

svmfit<-svm(wqs_data.yLBX~., svmdata,kernel ="radial")

svmfit

#Calculate shap for each row

s <- kernelshap(svmfit, svmdata[-35], bg_X = svmdata)

sv <- shapviz(s)

#Show overall shap

sv_importance(sv, kind = "both")

#Show the shap of individual 1

sv_waterfall(sv,row_id =1,max_display =2)

### 3.5.3 Decision Tree(DT)

#Install and load the R package

install.packages("rpart")

library(rpart)

#Perform decision tree regression.

rpdata<- data.frame(wqs_data[,1:34],wqs_data$yLBX)

rpfit<- rpart(wqs_data.yLBX~., data =rpdata)

rpfit

rpfit$cptable

plotcp(rpfit)

rpfit$variable.importance

barplot(rpfit$variable.importance)

#Calculate shap for each row

s <- kernelshap(rpfit, rpdata[-35], bg_X = rpdata)

sv <- shapviz(s)

#Show overall shap

sv_importance(sv, kind = "both")

#Show the shap of individual 1

sv_waterfall(sv,row_id =1,max_display =2)

### 3.5.4 Gradient Boosting Tree Model

#Install and load the R package

#install.packages("gbm")

library(gbm)

set.seed(123)

gbdata<- data.frame(wqs_data[,1:34],wqs_data$yLBX)

gbfit<- gbm(wqs_data.yLBX~.,data = gbdata,distribution = "gaussian")

gbfit

summary(gbfit)

## 3.6 Other machine learning algorithm variable screening

#Used in the caret package, it can mainly realize the importance of variables in the machine learning algorithm by the packaging method and filtering method.

#The variable selection in the mlr3 package also includes two types: filtering method and packaging method. However, the implementation method is slightly different from caret.

#Use random forest algorithm to display respectively

### 3.6.1 Variable screening of CARET random forest

#Install and load the R package

install.packages("randomForest")

install.packages("caret")

library(randomForest)

library(caret)

#modelling

set.seed(2345)

rfdata<- data.frame(wqs_data[,1:34],wqs_data$yLBX)

str(rfdata)

caretrf <- train(wqs_data.yLBX~., data = rfdata, method="rf")

#Variable importance display

install.packages("DALEX")

library(DALEX)

explainercaretrf <- explain(caretrf, label="rf", data = rfdata, y = rfdata$wqs_data.yLBX)

virf <- variable_importance(explainercaretrf, loss_function = loss_root_mean_square)

plot(virf)

control <- rfeControl(functions=rfFuncs, method="cv", number=10)

results <- rfe(rfdata[,1:34], rfdata[,35], sizes=c(1:34), rfeControl=control)

plot(results, type=c("g", "o"))

### 3.6.2 mlr3 package random forest variable screening

#Install and load the R package

install.packages("mlr3")

install.packages("mlr")

install.packages("mlrCPO") # mlrCPO contains some mlr preprocessing operations

devtools::install_github("mlr-org/mlr3learners")

install.packages("mlr3verse")

library(mlr)

library(mlrCPO)

library(mlr3learners)

library(mlr3)

library(mlr3verse)

mlrdata<-data.frame(wqs_data[,1:34],wqs_data$yLBX)

str(mlrdata)

task<-as_task_regr(mlrdata,target="wqs_data.yLBX")

#randomforest

learner<-lrn("regr.ranger")

learner$train(task)

learner$selected_features()

#Use filtering method criteria to extract features return values 1 (selected features) and 0 (deleted features)

filter<-flt("selected_features", learner = learner)

filter$calculate(task)

as.data.table(filter)

### 3.6.3 Bayesian variable selection method

#PIP indicates the relative importance of the impact on the outcome. The higher the value, the more important it is to the outcome.

ExtractPIPs(fit1)

##################

# 4. Methods for Identifying Multi-Exposure Interactions

## 4.1 Single factor analysis and linear model with interaction terms

#The interaction analysis of two variables is relatively simple, but the traditional method to test whether the interaction exists is to include the product term of the possible interaction variables in the model to see whether it is statistically significant.

#However, the statistical power of this analysis method is very low, especially when testing interactions between three or more pollutants, so in this example we only consider the first three pollutants in WQS.

lrdata<-data.frame(wqs_data[,1:3],wqs_data$ymultinomLBX)

str(lrdata)

lrdata$interaction1<-lrdata$LBX074LA*lrdata$LBX099LA

lrdata$interaction2<-lrdata$LBX074LA*lrdata$LBX105LA

lrdata$interaction3<-lrdata$LBX099LA*lrdata$LBX105LA

str(lrdata)

View(lrdata)

### 4.2 Two-Way ANOVA sample code

summary(aov(interaction1~wqs_data.ymultinomLBX, data =lrdata))

summary(aov(interaction2~wqs_data.ymultinomLBX, data =lrdata))

summary(aov(interaction3~wqs_data.ymultinomLBX, data =lrdata))

#visualization

boxplot(interaction1~wqs_data.ymultinomLBX, data = lrdata)

boxplot(interaction2~wqs_data.ymultinomLBX, data = lrdata)

boxplot(interaction3~wqs_data.ymultinomLBX, data = lrdata)

#Multiple classification logistic

#Install and load the R package

install.packages("nnet")

library(nnet)

llrfit <- multinom(wqs_data.ymultinomLBX ~ interaction1+interaction2+interaction3, data =lrdata)

summary(llrfit)

### 4.3 Bayesian Kernel Machine Regression(BKMR)

#You can view the multi-variable interaction analysis Predictor Response Bivar function

# 5. Methods for Nonlinear Effects

## 5.1 Restricted Cubic Spline(RCS)

library(rms)

# Fit restricted cubic spline, used here for variable x, similar to polynomial regression.

rcsdata<-data.frame(wqs_data[,1:3],wqs_data$yLBX)

str(rcsdata)

rcsfit<- lm(wqs_data.yLBX ~ rcs(LBX074LA,3)+LBX099LA+LBX105LA,data=rcsdata)

summary(rcsfit)

# Draw the original data

plot(rcsdata$LBX074LA,rcsdata$wqs_data.yLBX)

#Draw the fitting line

lines(rcsdata$LBX074LA, fitted(rcsfit),col="red")

# Draw fitting line

ggplot(rcsdata, aes(LBX074LA, wqs_data.yLBX) ) +

geom_point() +

stat_smooth(method =lm, formula = wqs_data.yLBX ~ rcs(LBX074LA,3)+LBX099LA+LBX105LA)

## 5.2 Generalized Additive Model (GAM)

#Install and load the R package

#install.packages("mgcv")

library(mgcv)g

library(splines)

# Build the model

gamdata<-data.frame(wqs_data[,1:3],wqs_data$yLBX)

str(gamdata)

gamfit<- gam(wqs_data.yLBX~ s(LBX074LA)+LBX099LA+LBX105LA, data = gamdata)

summary(gamfit)

#draw a picture and see

ggplot(gamdata, aes(LBX074LA, wqs_data.yLBX) ) +

geom_point() +

stat_smooth(method = gam, formula = wqs_data.yLBX~ s(LBX074LA)+LBX099LA+LBX105LA)

## 5.3 Quantile Regression (QR)

library(quantreg)

rqdata<-data.frame(wqs_data[,1:3],wqs_data$yLBX)

str(rqdata)

rqfit = rq(wqs_data.yLBX ~ LBX074LA, tau = c(0.05,0.25,0.5,0.75,0.95), data = rqdata)

plot(summary(rqfit))

## 5.4 Bayesian Kernel Machine Regression(BKMR)

#When the levels of the remaining variables are fixed at the median, the exposure-response cross-section of a single variable and the outcome mainly looks at the non-linear association between the exposure and the outcome.

#Predictor Response Univar() function

#Bivariate exposure dose-response curve: A dose-response curve of one variable with an outcome when the level of another variable is fixed at the percentile (the remaining variables are all fixed at the median).

#Predictor Response Bivar() function

## 5.5 Cross-Validated Kernel Ensemble (CVEK)

#Install and load the R package

install.packages("CVEK")

library(CVEK)

kern_par <- data.frame(method = c("linear", "rbf"),

l = rep(1, 2), p = 1:2, stringsAsFactors = FALSE)

#define kernel library

kern_func_list <- define_library(kern_par)

cvdata<-data.frame(wqs_data[,1:3],wqs_data$yLBX)

str(cvdata)

#Define reaction function

formula <- wqs_data.yLBX~ LBX074LA+ k(LBX099LA) + k(LBX105LA)

formula_test <- wqs_data.yLBX ~ k(LBX099LA):k(LBX105LA)

#Kernel integration for cross-validation based on the specified formula.

fit_bos <- cvek(formula, kern_func_list = kern_func_list, data = cvdata,

formula_test = formula_test,

lambda = exp(seq(-3, 5)), test = "asymp")

#Given the fitting object (fit_bos), the p value of the cvek test can be extracted as follows: P<0.05 means there is an interaction

fit_bos$pvalue

# first fit the alternative model

formula_alt <- wqs_data.yLBX~ LBX074LA+k(LBX099LA):k(LBX105LA)

fit_bos_alt <- cvek(formula = formula_alt, kern_func_list = kern_func_list,

data =cvdata, lambda = exp(seq(-3, 5)))

# Make predictions easier to interpret

LBX099LA_quantiles <- quantile(cvdata$LBX099LA, probs = c(.05, .25, .5, .75, .95))

LBX099LA_quantiles

LBX105LA<-cvdata$LBX105LA

# LBX099LA is set to its 5% quantile

data_test1 <- data.frame(cvdata,LBX105LA,

LBX099LA= LBX099LA_quantiles[1])

data_test1_pred <- predict(fit_bos_alt, data_test1)

# LBX099LA is set to its 25% quantile

data_test2 <- data.frame(cvdata, LBX105LA,

LBX099LA= LBX099LA_quantiles[1])

data_test2_pred <- predict(fit_bos_alt, data_test2)

# LBX099LA is set to its 50% quantile

data_test3 <- data.frame(cvdata, LBX105LA,

LBX099LA= LBX099LA_quantiles[1])

data_test3_pred <- predict(fit_bos_alt, data_test3)

# LBX099LA is set to its 75% quantile

data_test4 <- data.frame(cvdata, LBX105LA,

LBX099LA= LBX099LA_quantiles[1])

data_test4_pred <- predict(fit_bos_alt, data_test4)

# LBX099LA is set to its 95% quantile

data_test5 <- data.frame(cvdata, LBX105LA,

LBX099LA= LBX099LA_quantiles[1])

data_test5_pred <- predict(fit_bos_alt, data_test5)

# combine five sets of prediction data together

zpre <- rbind(data_test1_pred, data_test2_pred, data_test3_pred,

data_test4_pred, data_test5_pred)

str(zpre)

summary(cvdata)

LBX105LA_list <- seq(-3.1645,3.2896, length.out = 500)

data_pred <- data.frame(LBX105LA = rep(LBX105LA_list,5), zpre = zpre,

LBX099LA= rep(c("5% quantile", "25% quantile",

"50% quantile", "75% quantile",

"95% quantile"),each=500))

str(data_pred)

View(data_pred)

data_pred$LBX099LA<- factor(data_pred$LBX099LA,

levels = c("5% quantile", "25% quantile",

"50% quantile", "75% quantile",

"95% quantile"))

View(data_pred)

str(data_pred)

data_label <- data_pred[which(data_pred$LBX105LA ==3.2896 ), ]

data_label$value <- c("-1.6034860","-0.6543375","-0.0012800","0.6797575","1.7116680")

data_label$value <- factor(data_label$value, levels =

c("-1.6034860", "-0.6543375", "-0.0012800", "0.6797575", "1.7116680"))

library(ggplot2)

library(ggrepel)

ggplot(data = data_pred, aes(x = LBX105LA, y =zpre , color = LBX099LA)) +

geom_point(size = 0.1) +

geom_text_repel(aes(label = value), data = data_label,

color = "black", size = 3.6) +

scale_colour_manual(values = c("firebrick1", "chocolate2",

"darkolivegreen3", "skyblue2",

"white")) +

geom_line() + theme_set(theme_bw()) +

theme(panel.grid = element_blank(),

axis.title.x = element_text(size = 12),

axis.title.y = element_text(size = 12),

legend.title = element_text(size = 12, face = "bold"),

legend.text = element_text(size = 12)) +

labs(x = "LBX105LA",

y = "wqs_data.yLBX",

col = "per LBX099LA")
